# Rosacea and *Hippophae rhamnoides*: A Phytonutrient Approach to Skin Repair (The Systematic Review)

**DOI:** 10.3390/medicina62040676

**Published:** 2026-04-01

**Authors:** Maria Andrada Hincu, Doina Alina Nicoara, Elena Niculet, Lenuta Ambrose, Alin Laurentiu Tatu, Madalina Nicoleta Matei, Laura Bujoreanu-Bezman, Alin Codrut Nicolescu, Ticuta Negreanu-Pirjol, Sorin Tudorache

**Affiliations:** 1 Research Center of Morfofunctional Science, Faculty of Medicine and Pharmacy, “Dunarea de Jos” University of Galati, 800385 Galati, Romania; mariaandradaa99@gmail.com; 2 Faculty of Medicine, Ovidius University of Constanta, 900002 Constanta, Romania; alinadnicoara@yahoo.com; 3 Department of Morphological and Functional Sciences, Faculty of Medicine and Pharmacy, “Dunarea de Jos” University of Galati, 800385 Galati, Romania; 4 Multidisciplinary Integrated Centre of Dermatological Interface Research (MICDIR), “Dunarea de Jos” University of Galati, 800385 Galati, Romania; dralin_tatu@yahoo.com; 5 Department of Dermatology, “Saint Parascheva” Infectious Disease Clinical Hospital, 800179 Galati, Romania; 6 Clinical Medical Department, Faculty of Medicine and Pharmacy, “Dunarea de Jos” University of Galati, 800385 Galati, Romania; 7 Department of Medical Dentistry, Faculty of Medicine and Pharmacy, “Dunarea de Jos” University of Galati, 800385 Galati, Romania; madalinanmatei@yahoo.co.uk; 8 Surgical Medical Department, Faculty of Medicine and Pharmacy, “Dunarea de Jos” University of Galati, 800385 Galati, Romania; bezman.laura@yahoo.ro; 9 Medical Center “Roma” for Diagnosis and Treatment, 011773 Bucharest, Romania; nicolescualin66@yahoo.com; 10 Faculty of Medicine, “Carol Davila” University of Medicine and Pharmacy, 050474 Bucharest, Romania; 11 Egoclinic, District 1, 010235 Bucharest, Romania; 12 Faculty of Pharmacy, Ovidius University of Constanta, 900470 Constanta, Romania; ticuta.negreanu@univ-ovidius.ro; 13 Academy of Romanian Scientists, 3 Ilfov Street, 050044 Bucharest, Romania; 14 Faculty of Medicine, “Titu Maiorescu” University, 040441 Bucharest, Romania; sorin.tudorache@prof.utm.ro

**Keywords:** rosacea, *Hippophae rhamnoides*, sea buckthorn, inflammatory skin diseases, phytonutrients, skin barrier dysfunction, anti-inflammatory mechanisms

## Abstract

*Background and Objectives*: Rosacea is a chronic inflammatory dermatosis with a complex pathophysiology that continues to challenge effective long-term disease management. Rosacea is characterized by immune dysregulation, oxidative stress, neurovascular dysfunction, and impaired epidermal barrier integrity, while current therapeutic options remain limited. *Hippophae rhamnoides* (sea buckthorn) is a phytochemically rich medicinal plant with reported anti-inflammatory and dermo-reparative properties. *Materials and Methods*: A systematic literature review was conducted following PRISMA guidelines using the PubMed database. Studies published from 2020 onward evaluating sea buckthorn extracts, oils, or isolate bioactive compounds in the in vitro, in vivo, and limited clinical contexts were included, with emphasis on rosacea relevant mechanisms. *Results*: Twenty-six studies show that sea buckthorn compounds modulate inflammation, oxidative stress, vascular and immune responses, and barrier function in preclinical models. They consistently reduce pro-inflammatory mediators, improve barrier integrity, and attenuate immune or vascular activation, suggesting potential benefits for inflammatory skin disorders such as rosacea. *Conclusions*: *Hippophae rhamnoides* shows promising anti-inflammatory and skin-repairing effects that may benefit patients with rosacea. However, most evidence comes from preclinical studies, and clinical data specifically for rosacea are limited. Well-designed clinical trials are needed to confirm its effectiveness in treating this condition.

## 1. Introduction

Sea buckthorn (SB), also known as *Hippophae rhamnoides*, is a deciduous shrub rich in nutrients belonging to the family *Elaeagnaceae*, mostly found in coastal areas, river banks and mountainous zones in Eastern Europe and Asia [1]. SB has a long history as a therapeutic substance, being used to treat different skin-associated health problems (it was used for skin repair, UV-photoprotection, in psoriasis, allergic and atopic dermatitis, acne, ichthyosis) for thousands of years in several different cultures [2,3,3]. This paper examines the therapeutic potential of the phytochemical profile by relating it to the principal pathophysiological pathways implicated in inflammatory dermatological disorders. The biological effects of SB are mediated by modulating key molecular pathways: suppression of inflammation via down-regulation of NF-κB signaling, attenuation of oxidative stress through activation of the Nrf2/HO-1 pathway, and improvement of endothelial function by enhancing nitric oxide bioavailability. Its bioactive compounds (flavonoids, carotenoids, and unsaturated fatty acids) also contribute to immune regulation and protection against vascular dysfunction associated with chronic inflammation and oxidative stress [4,5]. The antioxidant composition differs between plant parts, contributing to the species’ documented biological, physiological, and medicinal benefits [6,7]. Scientific attention has concentrated on several key constituents, which are relevant in dermatological diseases and processes: ascorbic acid in berries, juice, and leaves; a range of carotenoids such as lycopene, lutein, zeaxanthin, *α*-carotene, *β*-carotene, and *γ*-carotene; tocopherols, notably *α*-, *β*-, and *γ*-tocopherol; abundant flavonoids like isorhamnetin and quercetin; and various polyphenols, with gallic acid predominant in leaves and berries and smaller amounts of caffeic, *p*-coumaric, and ferulic acids [8,9,10,11,12,13,14,15,16,17]. In dermatology, every part of SB can be used as follows: the seeds are used as oil for photoprotection, regeneration, burn wound reepithelization; the leaves for wound healing and recovery; fruit extracts used for UV-radiation-induced skin aging, acne, skin dehydration [3]. The bioactive profile of the plant reviewed above, rich in antioxidants, anti-inflammatory flavonoids and bioactive lipids suggests potential relevance to inflammatory cutaneous disorders. This relevance is particularly notable for rosacea, a prevalent inflammatory skin disorder, currently affecting over 5% of the world’s population, which translates in over 400 million people [18]. Recent studies support that rosacea is not merely a cosmetic condition but a chronic inflammatory dermatosis that may lead to ocular involvement, progressive vascular remodeling, phymatous tissue overgrowth, and significant psychosocial impairment [19]. To precisely assess the therapeutic potential of these naturally occurring compounds, the review prioritized articles that compared their effects with the established standard of care (described in detail in the following pages, together with the principal pathophysiological cascade), principally anti-inflammatory agents. The objective was to quantify the compounds’ pharmacological activity on the same molecular pathways targeted by conventional therapies and to evaluate both individual and combined effects relative to standard treatments. This paper advocates for pursuing affordable, widely accessible therapies by systematically exploring the association between the natural compound and the disease’s underlying mechanisms through the use of preclinical data studies and setting the goal for future clinical, patient-based research.

## 2. Materials and Methods

**Figure 1:**
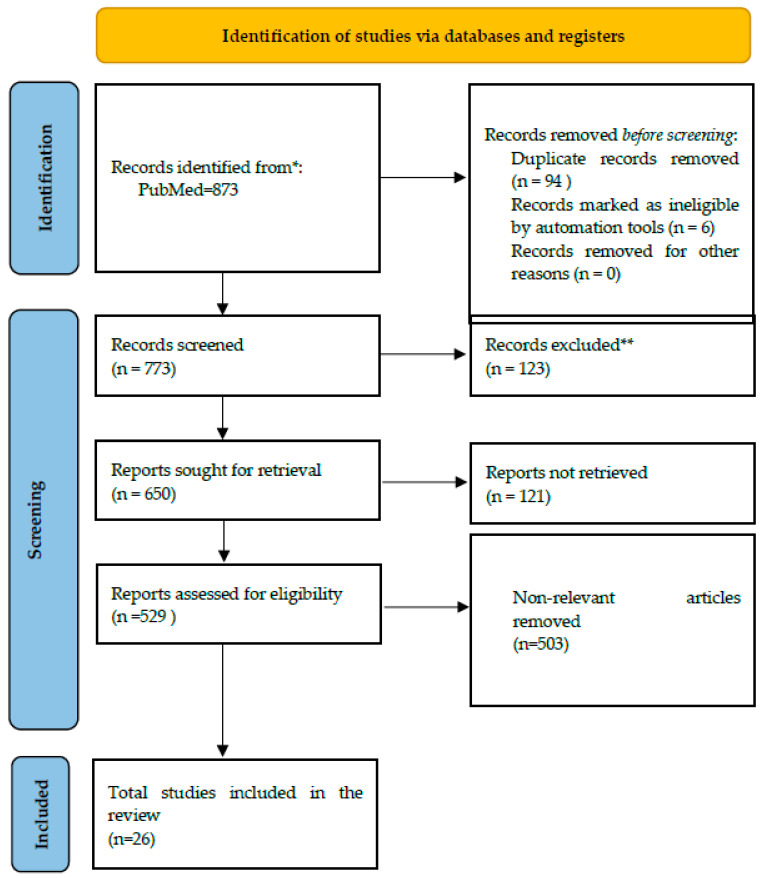
PRISMA 2020 flow diagram illustrating the identification, screening, eligibility, and inclusion of studies. * Consider, if feasible to do so, reporting the number of records identified from each database or register searched (rather than the total number across all databases/registers). ** If automation tools were used, indicate how many records were excluded by a human and how many were excluded by automation tools. Source: Page MJ, et al. 2021. Supplementary Material [20].

**Figure 2:**
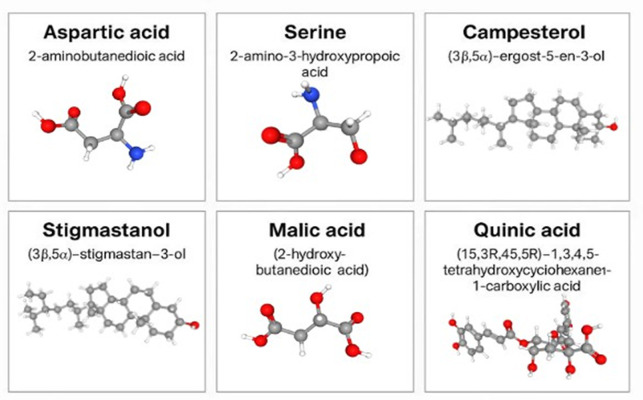
Bioactive constituents of sea buckthorn with potential relevance to inflammatory pathways. Common names and corresponding IUPAC nomenclature are shown, with designated areas for three-dimensional molecular conformations.

This review examines the therapeutic bioactive potential of compounds occurring naturally in SB, with particular emphasis on those noted above. The main objectives consisted of implementing PRISMA guidelines (visually represented in Figure 1) to identify, categorize, and synthesize the principal scientific literature, clarify and prioritize research gaps, and offer future perspectives on the compounds’ bioactivity in cutaneous inflammatory disorders. An important aim was to elucidate how these compounds modulate inflammatory diseases; we concentrated on the pathogenic mechanisms underlying rosacea. Rosacea was selected because its principal pathophysiological cascade (illustrated in Figure 2 and described in detail below) provides a representative framework for the development of therapeutic strategies. By analyzing how the compounds affect this cascade both individually and synergistically, we also aim to establish a rationale for accessible treatment approaches for the disorder. To identify relevant literature for this review, the PubMed database was queried, with the search completed on 22 November 2025. Broad search terms were employed to capture studies pertinent to the topics under consideration, with particular emphasis on investigations of molecular interactions between the compounds and human biological systems (Table 1). The keywords suggested directly by the databases were also considered. Published articles from 2020 onward were included, using PubMed’s “best match” filter. No language restrictions were imposed, since PubMed is a US-based publication website. During screening, original research and review articles were prioritized. Zotero was employed to import records and remove duplicates. After the screening process, the articles were fully read and categorized and their data have been presented in tables and figures, according to their type and content.

The purpose of this review was to address the following research question: “What evidence exists regarding the anti-inflammatory dermatological effects of SB, and through which pathophysiological mechanisms does it exert its effects in inflammatory skin diseases?”

1. The Population (P) primarily encompassed human participants diagnosed with rosacea, alongside relevant in vivo and in vitro experimental models designed to reflect the inflammatory and vascular pathophysiology of the condition.
2. The Intervention (I) consisted of *Hippophae rhamnoides* and its derivatives, including seed and pulp oils, plant extracts, and isolated bioactive compounds, administered through either topical or systemic routes.
3. The Comparator (C) included placebo, absence of intervention, or standard-of-care treatments, where applicable.
4. The Outcomes (O) focused on dermatological anti-inflammatory effects, with particular attention to predefined pathophysiological mechanisms of action. These encompassed modulation of pro- and anti-inflammatory cytokines, regulation of oxidative stress and inflammatory signaling pathways, reinforcement of skin barrier integrity, modulation of immune responses, regulation of vascular and neuroinflammatory processes, and promotion of tissue repair and wound-healing.

The initial search yielded 873 records. After removing 94 duplicate entries and 6 publications flagged as unidentified by the automation tool using Excel and Zotero(version 6), 773 articles remained for further review. A subsequent exclusion of 123 records that were not original research left 650 articles for eligibility assessment. The full-text retrieval process was applied to these articles, resulting in the exclusion of 121 studies whose complete texts were not publicly accessible. The remaining 529 articles were subsequently assessed for eligibility according to the criteria outlined above, which led to the further exclusion of 503 articles. Ultimately, a total of 26 studies were included in this paper, each demonstrating significant relevance to the topic under discussion. The current review was not registered and the protocol was not prepared.

## 3. Results

### 3.1. Contents of Hippophae rhamnoides

A visual presentation regarding the main phytoconstituents of the SB fruit extract (and their main dermatological roles) can be found in Table 2 [21,22,23,24,25,26,27,28]. *Hippophae rhamnoides* contains a wide spectrum of bioactive compounds, including phytosterols (Campesterol, Stigmastanol, and R-amyrin), carotenoids, polyunsaturated fatty acids, and organic acids such as α-linolenic acid [21,22]. Acting synergistically, these molecules modulate key inflammatory pathways, reduce oxidative stress, and support skin barrier and tissue repair, thereby contributing to meaningful relief in inflammatory conditions, both as standalone compounds and acting together [29,30,31,32]. A very important medicinal value is also brought up by the organic acids present in the SB. The predominant acids are malic and quinic, which together account for approximately 90% of the total fruit acids, although their concentrations vary across subspecies. In the principal genotypes, levels range between 4.2 and 6.5 g per 100 mL, a remarkably high concentration that is considered significant for the plant’s anti-inflammatory properties [33]. Sea buckthorn is a rich source of flavonoids, with isorhamnetin and quercetin identified as two of its major bioactive constituents. Quantitative chromatographic analyses have demonstrated that sea buckthorn berries contain substantial amounts of isorhamnetin derivatives, particularly isorhamnetin rutinoside, reported at approximately 40–50 mg/100 g fresh weight, while quercetin and its glycosides are present at lower but still biologically relevant concentrations, typically around 15–25 mg/100 g [34]. Comprehensive phenolic profiling studies further confirm that both isorhamnetin and quercetin represent dominant flavonol classes in sea buckthorn fruits and leaves, contributing significantly to its antioxidant and anti-inflammatory properties [33]. These findings are supported by RP-HPLC analyses, which consistently identify free and glycosylated forms of isorhamnetin and quercetin as characteristic components of *Hippophae rhamnoides* extracts.

Another important category of bioactive compounds in *Hippophae rhamnoides* is represented by amino acids, with the plant containing 18 of the 22 known amino acids [35]. These molecules serve as the fundamental building blocks of proteins, which are essential for cellular structure and function. In the context of inflammatory disorders, protein metabolism is frequently disrupted, resulting in impaired cellular division and compromised tissue regeneration. Adequate intake of these amino acids can therefore support the restoration of protein synthesis, enhance the healing capacity of the skin, and ultimately promote more effective tissue repair [36]. The precise concentrations of individual amino acids are provided in Table 3.

During the data extraction process, a recurring pattern was identified linking the molecular properties of several previously described compounds with their anti-inflammatory and tissue-restorative activities. The molecular structures of these compounds are presented in Figure 2 and are examined in detail in the following sections. The observed correlation sustains that the intake of these bioactive molecules is consistently associated with improved healing outcomes, thereby reinforcing the substantial therapeutic potential of *Hippophae rhamnoides* in the management of inflammatory disorders, with particular relevance to rosacea.

### 3.2. Inflammatory Cutaneous Pathophysiology of Rosacea

Rosacea is a chronic inflammatory skin disorder with a multifactorial and highly complex pathophysiology, in which molecular dysregulation of innate immunity, neurovascular signaling, and microbial interactions plays a central role.

At the molecular level, increased expression and activation of Toll-like receptor 2 (TLR2) in keratinocytes have been identified as key initiating events, leading to enhanced production and abnormal proteolytic processing of the antimicrobial peptide cathelicidin into proinflammatory fragments. These cathelicidin-derived peptides promote leukocyte chemotaxis, angiogenesis, and extracellular matrix degradation, thereby sustaining cutaneous inflammation and vascular remodeling characteristic of rosacea [37,38]. Concomitantly, upregulation of serine proteases, such as kallikrein 5, further amplifies inflammatory cascades and contributes to epidermal barrier dysfunction [39].

Neurovascular dysregulation represents an additional molecular axis in rosacea pathogenesis, involving altered expression of transient receptor potential (TRP) ion channels, including TRPV1 and TRPA1, on sensory nerves and keratinocytes. Activation of these channels by environmental triggers induces the release of vasoactive neuropeptides, such as substance P and calcitonin gene-related peptide, which drive vasodilation, increased vascular permeability, and neurogenic inflammation [40,41].

These molecular events explain the heightened vascular reactivity and persistent erythema observed in affected patients. Microbial factors further modulate molecular inflammatory pathways in rosacea. Increased density of Demodex mites has been associated with enhanced expression of inflammatory mediators, including matrix metalloproteinases and reactive oxygen species, which exacerbate tissue damage and vascular changes [42,43].

The presence of Demodex folliculorum in increased numbers within follicular openings in rosacea induces these changes, both directly and indirectly through its endosymbionts, and may also be involved in the ocular form of rosacea [43,44,45]. In parallel, alterations in the cutaneous and intestinal microbiome may influence systemic and local immune signaling, promoting chronic inflammation through cytokine-mediated pathways and reinforcing the concept of a gut–skin axis in rosacea [46,47].

**Figure 3:**
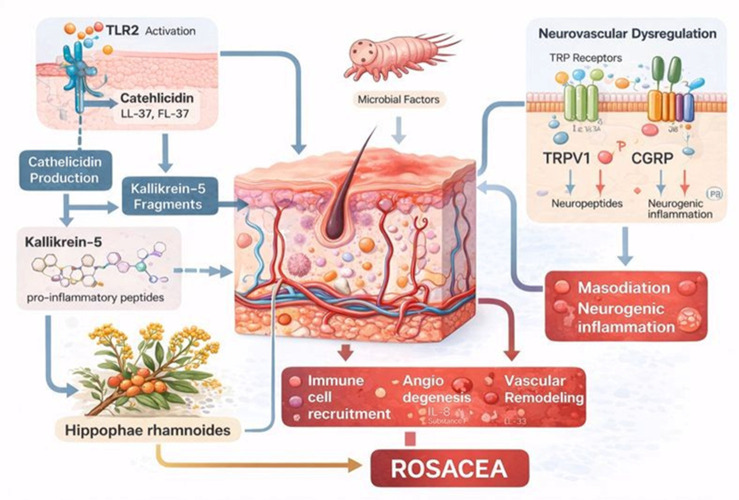
Schematic representation of the molecular pathophysiological cascade in rosacea, integrating innate immune activation (TLR2), dysregulated cathelicidin processing via kallikrein-5, microbial triggers (e.g., Demodex), and neurovascular dysregulation (TRP receptors, CGRP/neuropeptides), culminating in immune-cell recruitment, angiogenesis, and vascular remodeling. The diagram also highlights *Hippophae rhamnoides* as a putative modulatory component within this network.

Collectively, these molecular insights underscore rosacea as a disease driven by interconnected immunologic, neurovascular, and microbial signaling networks, providing a rationale for the development of targeted molecular therapies aimed at interrupting specific pathogenic pathways [48,49] (Figure 3).

### 3.3. Exploring the Bioactive Potential of Hippophae rhamnoides in Inflammatory Skin Disorders

The anti-inflammatory effects of SB are represented and organized in Table 4. Most of the available research is focused on the direct outcomes of different compounds found in SB within the context of inflammatory disease. The full pathophysiological cascade of rosacea is not yet fully understood [40,50,51]. However, stimulation or inhibition of key points within the known inflammatory cascade has been associated with improved therapeutic outcomes [40]. Animal in vivo studies represent the most common experimental design. This must be taken into careful consideration when conclusions are directly applied to clinical contexts.

Currently, the literature does not provide a highly in-depth analysis. Nevertheless, there is substantial evidence suggesting that SB may represent a strong candidate for future disease management. Standardization of SB extracts and administration protocols is lacking, whereby this limitation hinders reproducibility and clinical translation. Future research should focus on large-scale controlled clinical trials and long-term toxicity studies. These limitations do not diminish the potential of SB in the management of inflammatory skin disorders particularly rosacea. The compounds listed above have frequently been reported in the current literature as effective contributors to an improved standard of care in various inflammatory conditions. For this reason, the table below includes both the identified compounds and their associated mechanisms of action within the inflammatory context. Presenting this information in a structured manner allows for clearer interpretation of existing evidence, while also highlighting shared, complementary, or overlapping biological pathways. Through this approach, we aim to provide a meaningful starting point for future research specifically focused on this topic. Such investigations may enable a more nuanced exploration of molecular mechanisms and therapeutic interactions, ultimately supporting not only a better understanding of these complex pathologies, but also the identification of accessible and affordable therapeutic strategies based on bioactive compounds already found in nature.

## 4. Discussion

This review synthesizes recent evidence regarding the dermatological and anti-inflammatory potential of *Hippophae rhamnoides*, with a particular focus on mechanisms relevant to rosacea and related inflammatory skin disorders. The studies summarized in Table 1 collectively reveal several consistent mechanistic patterns that support the biological plausibility of sea buckthorn-based interventions in chronic cutaneous inflammation. A dominant and recurring theme across the included studies is the central role of inflammatory signaling pathways regulated by nuclear factor kappa B. Multiple experimental investigations demonstrate that both whole extracts and isolated compounds derived from *Hippophae rhamnoides* suppress nuclear factor kappa B activation, leading to reduced expression of pro-inflammatory cytokines and mediators [60,61,62,65]. This mechanism is consistently observed across diverse biological systems, including keratinocytes, macrophages, endothelial cells, mast cells, and epithelial barrier models. Importantly, one of the studies directly addressing rosacea shows that quercetin interacts with key regulators of nuclear factor kappa B signaling, resulting in attenuation of immune infiltration and vascular dysfunction [53]. The convergence of these findings across different disease models suggests that modulation of nuclear factor kappa B signaling represents a core anti-inflammatory mechanism shared by multiple sea buckthorn-derived constituents. Another clear pattern emerging from Table 1 is the simultaneous modulation of inflammation and epithelial barrier integrity. Several studies demonstrate that sea buckthorn oils, polysaccharides, and flavonoids not only suppress inflammatory cytokine production but also restore structural and functional components of epithelial barriers, including tight junction proteins and tissue architecture [40,43,46,48]. This dual effect is particularly relevant to rosacea, where impaired barrier function is increasingly recognized as a contributor to disease chronicity and sensitivity. The ability of sea buckthorn constituents to improve barrier integrity while dampening inflammation suggests a mechanism that may support sustained clinical improvement rather than transient symptom control. Analysis of the table also reveals a distinction between the effects of isolated bioactive compounds and whole-plant preparations. Isolated flavonoids such as quercetin, isorhamnetin, and 1,5-dimethyl citrate primarily exert targeted molecular effects, including inhibition of transcription factors, inflammatory enzymes, and intracellular signaling kinases [49,50,52,53,76]. In contrast, whole extracts and oils derived from berries, seeds, or leaves consistently demonstrate broader biological activities, including antioxidant defense enhancement, lipid barrier restoration, angiogenesis modulation, and promotion of tissue regeneration [42,43,45,51,77]. This pattern suggests that synergistic interactions among multiple phytochemical classes may underlie the more comprehensive dermatological benefits observed with complex sea buckthorn formulations.

A further important observation from Table 1 is the recurrent involvement of vascular and endothelial mechanisms, which are particularly relevant to rosacea pathophysiology. Several studies report reduced angiogenesis, endothelial activation, and adhesion molecule expression following treatment with sea buckthorn-derived compounds [47,53]. Given that persistent erythema and abnormal microvascular responses are hallmark features of rosacea, these findings provide a mechanistic link between experimental observations and clinical manifestations of the disease. Despite the overall consistency of mechanistic findings, Table 1 also highlights several limitations in the current evidence base. There is substantial heterogeneity in extract composition, dosing regimens, and routes of administration across studies [40,42,43,44,45,46,47,48,49,50,51,52,53,54]. Additionally, the majority of mechanistic insights are derived from in vitro or animal models, underscoring the need for more standardized and disease-specific clinical investigations. The relative scarcity of rosacea-focused clinical trials represents a key gap that should be addressed in future research. In summary, the evidence summarized in Table 1 [40,42,43,44,45,46,47,48,49,50,51,52,53,54] indicates that *Hippophae rhamnoides* exerts its dermatological effects through a multi-target mode of action, simultaneously modulating inflammatory signaling, immune responses, oxidative stress, vascular dysfunction, and epithelial barrier integrity. This integrated mechanism differentiates sea buckthorn from single-target therapeutic approaches and supports its potential role as an adjunctive or integrative strategy in the management of rosacea and other inflammatory skin disorders.

### 4.1. Future Perspectives

Although the evidence summarized in Table 1 consistently supports the anti-inflammatory and dermatological potential of SB, the current stage of research remains predominantly exploratory. Most available data originate from in vitro experiments, animal models, or limited clinical contexts, which restrict direct translation to human inflammatory skin diseases such as rosacea. A critical priority for future research is the standardization of *Hippophae rhamnoides* extracts. Across the included studies, considerable heterogeneity exists with respect to plant parts used, extraction procedures, phytochemical composition, and administered doses. This lack of standardization limits reproducibility and prevents meaningful comparison between studies. Future investigations should therefore focus on developing well-characterized extracts with defined phytochemical profiles to ensure consistency across experimental platforms. From a translational perspective, future research should follow a stepwise strategy. Large scale and rigorously designed in vivo studies should be prioritized to confirm efficacy, safety, and dose–response relationships under standardized conditions. These studies should subsequently be complemented by mechanistic in vitro investigations aimed at validating molecular targets, clarifying signaling pathways, and identifying potential synergistic interactions between different classes of bioactive compounds. Only if preclinical findings remain robust and reproducible should progression toward human studies be considered. At that stage, carefully designed clinical trials with clearly defined endpoints, adequate sample sizes, and disease-specific stratification will be essential. In particular, rosacea-focused clinical studies remain underrepresented within the current literature and should constitute a major focus of future research efforts, with clearly stated end-points such as erythema scores, lesion counts, barrier function, or patient-reported outcomes. Rosacea is currently understood as a multifactorial disorder with interactions between the immune, nervous and vascular systems, disease exacerbation being driven by neuroimmune and inflammatory pathways, with environmental factors’ involvement. The skin can be seen as a neuro-immune and endocrine organ current literature providing a framework in order to understand how external stimuli can activate networks involving cytokines, neuropeptides, and hormonal mediators. The neuro-immune and endocrinological link to rosacea will be integrated through future research, establishing their contribution to disease onset, progression and therapeutic response [78].

### 4.2. Clinical Implications and Limitations

Despite the promising biological effects observed in experimental models, the current body of evidence does not support the clinical use of *Hippophae rhamnoides* as a standalone therapeutic intervention for the treatment of rosacea or other inflammatory skin diseases. As summarized in Table 1, the majority of studies are conducted at a preclinical or early translational level and do not meet the criteria required for routine clinical application. At present, the absence of standardized formulations, limited large-scale in vivo validation, and lack of robust rosacea-specific clinical trials represent major barriers to clinical translation. Furthermore, variability in extract composition and dosing raises concerns regarding reproducibility, safety, and interindividual variability in therapeutic response. These limitations highlight the risks associated with premature clinical adoption. From a clinical standpoint, *Hippophae rhamnoides* should therefore be regarded as an experimental or investigational candidate rather than an established therapeutic option. Current findings are valuable for hypothesis generation and future trial design, but they are insufficient to inform clinical decision-making. If future studies succeed in establishing standardized extracts, confirming efficacy and safety in large-scale preclinical models, and subsequently demonstrating benefit in well-designed human trials, *Hippophae rhamnoides* may eventually emerge as a supportive intervention in inflammatory skin disorders. Until such evidence becomes available, its role should remain confined to the research setting. Therapeutic potential is evident; however, further investigation is needed to clarify the precise pharmacological and therapeutic mechanisms.

## 5. Conclusions

This review integrates recent experimental and translational findings examining the dermatological and anti-inflammatory properties of *Hippophae rhamnoides*. As summarized in Table 1, available studies suggest that sea buckthorn derived compounds can influence several biological processes implicated in inflammatory skin disorders, including immune signaling, oxidative stress responses, vascular regulation, and epithelial barrier function. A consistent observation across experimental models is the modulation of inflammatory and barrier-related pathways by sea buckthorn constituents. Multiple studies report reductions in pro-inflammatory mediators, partial restoration of barrier integrity, and attenuation of vascular or immune activation. These recurring mechanistic patterns provide a plausible biological basis for the reported effects in preclinical models, including those with relevance to rosacea-associated pathology. Nevertheless, the current evidence base is predominantly preclinical and methodologically heterogeneous. Substantial variability in extract composition, dosing regimens, experimental systems, and outcome measures limits comparability across studies and constrains the strength of translational inferences. Moreover, direct clinical evidence, particularly rosacea-specific human data, remains sparse or absent, precluding firm conclusions regarding therapeutic efficacy.

In summary, *Hippophae rhamnoides* demonstrates measurable biological activity in experimental models relevant to inflammatory skin disease, but its clinical utility has not yet been established. Further investigation using standardized formulations, clearly defined endpoints, and well-designed clinical studies is required to determine whether these preclinical findings translate into a meaningful therapeutic benefit.

## Figures and Tables

**Table 1:** Search terms used for database queries (PubMed).

Stand-Alone Search Terms	General Terms for Combinations	Suggested Mechanistic Terms
Rosacea	Inflammatory skin disease; chronic inflammation; vascular disorders	TLR2; LL-37; TRPV1; angiogenesis; Demodex
*Hippophae rhamnoides*/Sea Buckthorn	medicinal plant; plant extracts; nutraceuticals	berry oil; CO_2_ extract; palmitoleic acid (*ω*-7)
Carotenoids	photoprotection; oxidative stress; epithelial repair	lycopene; *β*-carotene; lutein; VEGF signaling
Polyphenols/ Flavonoids	Antioxidant activity; immune modulation	quercetin; isorhamnetin; NF-*κ*B; MAPK
PUFAs	barrier repair; resolution of inflammation	*ω*-7; linoleic acid; TNF-*α*; IL-6 pathways
Vitamin C	collagen synthesis; extracellular matrix stabilization	ECM remodeling; reduction in persistent erythema
Tocopherols (Vitamin E)	lipid peroxidation control; membrane protection	*α*-tocopherol; oxidative flare attenuation
Zinc	immune modulation; epithelial proliferation	keratinocyte repair; antioxidant enzyme cofactor

**Table 2:** Bioactive compounds in sea buckthorn and their relevance to skin health and rosacea.

Phytochemical Class	Dermatological and Anti-Rosacea Roles	Ref.
Carotenoids	Antioxidant protection against reactive oxygen species; reduce photo-induced vascular damage; support collagen synthesis and epithelial regeneration; may reduce persistent erythema.	[21].
Polyunsaturated fatty acids (PUFAs)	Restore epidermal lipid organization and reduce transepidermal water loss (TEWL); downregulate pro-inflammatory cytokines including IL-6 and TNF-*α* involved in neurovascular dysregulation in rosacea; support post-inflammatory barrier repair.	[22].
Phytosterols	Improve cutaneous microcirculation; modulate inflammatory mediators; support vascular stability in erythematous subtypes; promote recovery following irritation.	[23].
Tocopherols (Vitamin E)	Protect membrane lipids from peroxidation; reduce inflammation-related discomfort; attenuate oxidative pathways that prolong flare duration.	[24].
Vitamin C	Enhances collagen remodeling and extracellular matrix repair; stabilizes dermal vasculature; reduces oxidative stress contributing to telangiectasia and facial redness.	[24].
Polyphenolic compounds	Exert antioxidant and anti-inflammatory effects; modulate NF-*κ*B and MAPK signaling implicated in cutaneous immune dysregulation; promote wound healing.	[25].
Organic acids	Enhance dermal perfusion and acid-mantle defense; support tissue repair and resolution of irritation; may contribute to antimicrobial function.	[22].
Vitamin B complex	Supports nerve recovery and cellular regeneration; may reduce neurogenic hypersensitivity contributing to flushing in rosacea.	[26].
Vitamin K	Strengthens microvascular integrity; reduces capillary fragility and persistent facial erythema.	[26].
Coumarins and triterpenes	Modulate inflammatory signaling; may reduce stress-triggered vasodilation; support neuromodulatory balance.	[27].
Zinc	Supports keratinocyte proliferation and barrier restoration; cofactor for antioxidant enzymes; may reduce pustular lesions and enhance vitamin A utilization.	[28].

**Table 3:** Amino acids found in sea buckthorn [35].

Amino Acid	Content (mg/100 g)
Aspartic acid	426.6
Serine	28.1
Glutamine	19.4
Glycine	16.7
Alanine	21.2
Cysteine	3.3
Valine	21.8
Ammonia	41.8
Tyrosine	13.4
Isoleucine	17.4
Methionine	2.3
Proline	45.2
Phenylalanine	20.0
Histidine	13.7
Lysine	27.2
Threonine	36.8
Arginine	11.3

**Table 4:** Summary of included studies.

Pathogenic Mechanism	Mechanism of Action	Type of Study	Conclusions	Study
A.				
Atopic dermatitis is driven by immune dysregulation with dominance of T helper 2 responses, increased immunoglobulin E production, mast cell infiltration, excessive cytokine release, and impaired skin barrier function.	Regulates the balance between T helper 1 and T helper 2 immune responses by reducing inflammatory cytokine production, serum immunoglobulin E levels, and inhibiting migration and maturation of Langerhans cells.	In vivo mouse model of chemically induced atopic dermatitis.	Sea buckthorn oil attenuates atopic dermatitis-like skin inflammation by immunomodulation and suppression of T helper 2 dominant responses.	[52].
Inflammation is driven by activation of macrophages leading to excessive production of nitric oxide, pro-inflammatory cytokines, reactive oxygen species, and mitochondrial dysfunction.	The triterpenes oleanolic acid, asiatic acid, and maslinic acid suppress inflammatory responses by inhibiting inflammatory mediator production, oxidative stress, and intracellular signaling pathways involved in inflammation.	In vitro experimental study using a murine macrophage cell inflammation model.	Oleanolic acid, asiatic acid, and maslinic acid exhibit significant anti-inflammatory effects in macrophages, supporting their potential use as natural anti-inflammatory agents.	[53].
B.				
Cellular inflammation is characterized by increased production of pro-inflammatory cytokines driven by oxidative stress and accumulation of toxic lipid-associated contaminants.	Refining of SB pulp oil increases anti-inflammatory activity by enhancing tocopherol content, improving oxidative stability, and reducing the production of pro-inflammatory cytokines in intestinal epithelial cells.	In vitro experimental study using human intestinal epithelial cell culture and physicochemical analysis of refined sea buckthorn pulp oil.	Appropriate refining improves oxidative stability and cellular anti-inflammatory potential of sea buckthorn pulp oil by increasing beneficial micronutrients and reducing harmful compounds.	[54].
Immune-mediated liver injury is characterized by excessive activation of inflammatory signaling pathways, increased tumor necrosis factor α and interleukin 1 β production, nitric oxide-mediated oxidative and nitrative stress, and down-regulation of hepatic drug-metabolizing enzymes.	SB extract suppresses nuclear factor kappa B signaling, reduces inflammatory cytokine production and inducible nitric oxide synthase expression, and restores cytochrome P450 3A expression and metabolic activity in the liver.	In vivo experimental rat study of immune-mediated liver injury induced by immune stimulation.	Sea buckthorn protects liver function by inhibiting inflammatory signaling and preventing the down-regulation of hepatic drug-metabolizing enzymes during immune-mediated liver injury.	[55].
Immune-mediated liver injury leads to suppression of cytochrome P450 2D6 expression through inflammatory cytokine signaling, reduced cyclic adenosine monophosphate activity, and sustained activation of nuclear factor kappa B, resulting in impaired hepatic drug metabolism.	*Hippophae rhamnoides* reverses the decrease in hepatic cytochrome P450 2D6 by increasing cyclic adenosine monophosphate levels, activating protein kinase A, inhibiting nuclear factor kappa B activity, and restoring transcriptional regulation of drug-metabolizing enzymes.	In vivo experimental rat study investigating metabolic enzyme regulation during immune-mediated liver injury.	*Hippophae rhamnoides* restores cytochrome P450 2D6 dependent drug metabolism during immune-mediated liver injury by modulating inflammatory and cyclic adenosine monophosphate dependent signaling pathways.	[56].
C.				
Colitis is driven by chronic intestinal inflammation associated with oxidative stress, disruption of intestinal barrier integrity, and gut microbiota dysbiosis characterized by reduced short-chain fatty acid-producing bacteria.	SB polysaccharide reduces intestinal inflammation and oxidative stress while improving intestinal barrier function by modulating gut microbiota composition and increasing short-chain fatty acid production.	In vivo experimental mouse study of chemically induced colitis with fecal microbiota transplantation.	Sea buckthorn polysaccharide alleviates colitis by restoring gut microbiota balance, increasing short-chain fatty acids, and improving intestinal inflammation and barrier dysfunction.	[57].
Interstitial cystitis is driven by chronic bladder inflammation associated with oxidative stress, mast cell activation, fibrosis, apoptosis, and impaired bladder wall integrity following chemical injury.	SB reduces bladder inflammation, mast cell infiltration, fibrosis, and apoptosis through antioxidant and anti-inflammatory effects that limit oxidative damage and inflammatory tissue remodeling.	In vivo experimental rat study of chemically induced cystitis compared with standard oral therapy.	Sea buckthorn significantly alleviates bladder inflammation and tissue damage with efficacy comparable to conventional therapy, supporting its potential as a supplementary treatment for interstitial cystitis.	[58].
Rosacea is a chronic inflammatory skin disorder driven by immune dysregulation, neurovascular dysfunction, oxidative stress, and impaired skin barrier function, leading to persistent erythema and inflammatory lesions.	Vitamins and nutrients modulate inflammatory pathways, immune responses, vascular reactivity, and skin barrier integrity, thereby reducing inflammation, erythema, and lesion severity in rosacea.	Narrative review of clinical and experimental studies on vitamins and nutrients in ro-sacea management.	Vitamins and nutrients represent safe and cost-effective adjunctive options for rosacea management, although further well-designed clinical trials are required to establish standardized supplementation recommendations.	[59].
D.				
Acne and rosacea are influenced by diet-related metabolic and inflammatory mechanisms, including insulin-like growth factor 1 signaling, immune dysregulation, oxidative stress, and neurovascular reactivity, which modulate disease risk and severity.	Dietary patterns consistent with a Mediterranean-style diet reduce inflammatory and metabolic risk factors by limiting insulin-like growth factor 1 activation, decreasing pro-inflammatory signaling, and supporting skin barrier and immune homeostasis.	Cross-sectional controlled clinical study comparing acne and rosacea patients with matched healthy control groups.	Diet significantly influences acne and rosacea risk and severity, and disease-specific dietary scores may support personalized nutritional counseling as part of clinical management.	[60].
Rosacea is driven by neurovascular dysregulation associated with metabolic abnormalities, particularly elevated circulating amino acids that enhance vasodilation and vascular reactivity rather than primary inflammatory cell infiltration.	Aberrant amino acid metabolism, especially increased glutamic acid and aspartic acid, promotes neurovascular reactivity by stimulating nitric oxide production in endothelial cells and keratinocytes and inducing vasodilation-related neuropeptide release from peripheral neurons.	Combined clinical metabolomics study with in vivo mouse experiments and in vitro cellular mechanistic analyses.	Dysregulated amino acid metabolism is a key driver of neurovascular reactivity in rosacea, identifying metabolic pathways as potential therapeutic targets for disease management.	[61].
Papulopustular rosacea severity is associated with metabolic and inflammatory dysregulation, including elevated serum homocysteine levels, reduced vitamin B12 and folic acid levels, oxidative stress, impaired skin barrier function, and increased Demodex density.	Elevated homocysteine promotes oxidative stress and inflammatory cytokine signaling, leading to endothelial dysfunction, increased transepidermal water loss, enhanced Demodex proliferation, and aggravation of inflammatory skin lesions.	Case-control clinical study comparing papulopustular rosacea patients with healthy controls.	Serum homo- cysteine levels correlate positively with disease severity, while vitamin B12 and folic acid deficiencies are associated with more severe papulopustular rosacea, suggesting a contributory role of metabolic imbalance in disease pathogenesis.	[62].
E.				
Inflammatory skin diseases such as psoriasis, atopic dermatitis, rosacea, and wounds are driven by immune dysregulation, oxidative stress, impaired skin barrier function, and altered lipid composition, leading to chronic inflammation and delayed tissue repair.	SB oil modulates inflammatory pathways, reduces oxidative stress, enhances skin barrier integrity, and promotes epithelial regeneration through its rich content of omega fatty acids, vitamins, antioxidants, and bioactive phytochemicals.	Narrative review of clinical, in vivo, and in vitro studies evaluating sea buckthorn oil in inflammatory and barrier-related skin diseases.	Sea buckthorn oil shows promise as a safe adjunctive therapy for inflammatory skin conditions by improving inflammation control, skin barrier repair, and wound healing, although formulation challenges and larger clinical trials remain necessary.	[63].
Chronic skin and mucosal inflammatory diseases are driven by persistent immune activation, oxidative stress, dysregulated inflammatory signaling cascades, impaired barrier integrity, and defective tissue repair processes.	SB and its bioactive compounds exert multi-target anti-inflammatory effects by activating endogenous antioxidant defenses, suppressing pro-inflammatory signaling pathways, modulating immune cell responses, and promoting tissue repair and barrier restoration across skin and mucosal tissues.	Narrative review integrating in vitro, in vivo, and clinical studies on sea buckthorn in skin and mucosal inflammation.	Sea buckthorn represents a promising multi-target natural therapeutic for inflammatory skin and mucosal diseases, although further standardized formulations and high-quality clinical trials are required to support clinical translation.	[3].
Psoriasis is driven by chronic immune-mediated skin inflammation characterized by oxidative stress, dendritic cell activation, and pathological expansion of T helper 1 and T helper 17 cells with excessive pro-inflammatory cytokine production.	Isorhamnetin attenuates psoriasiform skin inflammation by reducing oxidative stress, inhibiting nuclear factor kappa B signaling, suppressing dendritic cell maturation, and decreasing T helper 1 and T helper 17 cell differentiation and cytokine release.	In vivo experimental mouse study using an imiquimod-induced psoriasiform dermatitis model.	Isorhamnetin significantly alleviates psoriasis-like skin lesions by targeting oxidative stress and immune dysregulation, supporting its potential as an immunomodulatory treatment for inflammatory skin diseases.	[64].
F.				
Type I allergic reactions are driven by immunoglobulin E-mediated mast cell activation, resulting in intracellular Ca^2+^ influx, phosphorylation of spleen tyrosine kinase, and excessive release of chemical mediators such as histamine and leukotriene B4.	Polyphenol-rich fractions from SB berries, particularly isorhamnetin, inhibit mast cell activation by suppressing Ca^2+^ influx, reducing phosphorylation of spleen tyrosine kinase, and decreasing histamine and leukotriene B4 release.	In vitro experimental study using rat basophilic leukemia cells and mouse mast cell lines.	Sea buckthorn berry polyphenols exert potent anti-allergic effects by targeting early intracellular signaling events in mast cells, supporting their potential use in managing allergic and inflammatory skin conditions.	[65].
Second-degree burn wounds are characterized by acute inflammation, tissue damage, risk of infection, and delayed epithelial regeneration, leading to prolonged healing time and increased care burden.	SB cream accelerates wound healing by reducing local inflammation and promoting tissue repair and re-epithelialization, resulting in faster restoration of skin integrity compared with silver sulfadiazine treatment.	Randomized triple-blind clinical trial comparing sea buckthorn cream with silver sulfadiazine dressing in patients with second-degree burns.	Sea buckthorn cream significantly shortens the healing period of second-degree burn wounds and demonstrates superior clinical efficacy compared with silver sulfadiazine, suggesting a beneficial role in burn wound management.	[66].
Third-degree burn wounds have severe tissue destruction, prolonged inflammation, oxidative stress, impaired angiogenesis, disrupted dermal hydration, reduced collagen deposition, and altered cellular bioenergetics, leading to delayed wound repair.	Dual treatment: photobio-modulation—904 nm superpulsed laser and SB leaf extract, reduces oxidative stress + inflammatory cyto-kine production, activates nuclear factor erythroid 2 –related factor 2 signaling, enhances angiogenesis, collagen synthesis, dermal hydration, and restores cellular bioenergetics to surge wound repair.	In vivo trial rat study of third-degree burn wound healing comparing photobiomodulation, *Hippophae rhamnoides* leaf extract, and combination therapy.	The combined photobiomodulation and *Hippophae rhamnoides* treatment synergistically accelerates third-degree burn wound healing by targeting inflammation, oxidative stress, angiogenesis, extracellular matrix deposition, and cellular energy metabolism.	[67].
G.				
Skin aging and inflammatory skin changes are associated with oxidative stress, increased pro-inflammatory cytokine production, impaired lipid barrier integrity, reduced collagen synthesis, and microvascular dysfunction leading to dryness, redness, and loss of elasticity.	Oral SB oil supplementation enhances antioxidant defenses by increasing catalase activity, reduces tumor necrosis factor alpha levels, improves lipid-mediated skin barrier function, and promotes collagen synthesis, resulting in improved skin hydration, elasticity, texture, and reduced redness.	Randomized double-blind placebo-controlled clinical trial evaluating oral sea buckthorn oil supplementation in healthy adult women (12-weeks).	Oral sea buckthorn oil significantly improves skin barrier function, reduces inflammatory + oxidative stress markers, and enhances skin hydration, elasticity, and collagen content, supporting its role as a functional nutritional approach for inflammatory and aging-related skin conditions.	[68].
Rosacea is driven by immune and vascular dysfunction characterized by inflammatory cell infiltration, pathological angiogenesis, endothelial activation, and sustained nuclear factor kappa B signaling leading to chronic cutaneous inflammation.	Quercetin alleviates ro-sacea by directly binding to p65 and intercellular adhesion molecule 1, suppressing nuclear factor kappa B signaling, reducing inflammatory mediator expression in keratinocytes and endothelial cells, and inhibiting immune infiltration and angiogenesis.	Integrated analysis combined with in vivo rosacea-like mouse models and in vitro experiments in human keratinocytes and dermal microvascular endothelial cells.	It reduces inflammatory and vascular dysfunction in rosacea by direct molecular targeting of p65 and intercellular adhesion molecule 1, supporting it as a novel therapeutic compound for rosacea.	[34].
Alteration of the cutaneous microbiome associated with *Demodex folliculorum*, with isolation of *Bacillus cereus* in patients with topical steroid-induced rosaceiform facial dermatitis, suggesting a microorganism-mediated inflammatory mechanism.	Topical corticosteroid misuse promotes *Demodex* proliferation and microbial overgrowth; *Bacillus cereus* may act as a pro-inflammatory trigger through toxin production and immune activation, contributing to rosaceiform inflammation.	Observational clinical study with microbiological isolation and identification.	A microbiome-driven pathogenic pathway in topical steroid-induced rosaceiform facial dermatitis; it emphasizes the role of *Demodex*—associated bacteria as contributors to inflammation and therapeutic targets.	[69].
H.				
Inflammatory responses are driven by lipopolysaccharide-induced activation of macrophages, leading to excessive nitric oxide production, upregulation of inducible nitric oxide synthase and cyclooxygenase-2, increased pro-inflammatory cytokine release, and sustained nuclear factor kappa B signaling.	1,5-Dimethyl citrate isolated from SB fruits suppresses inflammation by inhibiting IB kinase activation, preventing nuclear factor kappa B p65 phosphorylation and nuclear translocation, downregulating inducible nitric oxide synthase and cyclooxygenase-2 expression, and reducing interleukin-6 and tumor necrosis factor alpha production.	In vitro experimental study.	1,5-Dimethyl citrate from Hippophae rhamnoides exhibits potent anti-inflammatory activity through inhibition of the nuclear factor kappa B signaling pathway, supporting its potential application in the management of inflammatory diseases involving macrophage-mediated immune responses.	[70].
Inflammatory and degenerative skin disorders are driven by immune dysregulation, oxidative stress, microbial colonization, impaired skin barrier function, and delayed tissue regeneration, leading to chronic inflammation, wounds, and dermatological lesions.	SB exerts dermatological benefits through its rich content of flavonoids, carotenoids, vitamins, polyunsaturated fatty acids, and antioxidants that collectively reduce inflammation, inhibit microbial growth, modulate sebum production, enhance skin barrier integrity, and promote wound healing and tissue regeneration.	Narrative review of ethnomedicinal uses, phytochemistry, pharmacological studies, and dermatological applications of *Hippophae rhamnoides*.	Ethnomedicinal and pharmacological evidence supports the use of *Hippophae rhamnoides* in inflammatory and infectious skin diseases, wound healing, burns, and cosmeceutical applications, although further well-controlled clinical studies are required.	[71].
I.				
Atopic dermatitis is characterized by immune dysregulation involving imbalanced T helper 1 and T helper 2 responses, increased production of pro-inflammatory cytokines, mast cell infiltration, and impaired skin barrier function associated with reduced filaggrin expression.	Total flavonoids of SB ameliorate atopic dermatitis by suppressing nuclear factor kappa B and mitogen-activated protein kinase signaling, reducing pro-inflammatory cytokine production, inhibiting mast cell infiltration, restoring filaggrin expression, and rebalancing T helper 1 and T helper 2 immune responses.	Combined in vivo and in vitro experimental study.	Topical administration of total flavonoids of *Hippophae rhamnoides* significantly improves atopic dermatitis-like skin lesions by reducing inflammation and repairing skin barrier dysfunction, supporting their potential as a topical therapeutic candidate for atopic dermatitis.	[72].
Skin inflammation and damage are driven by ultraviolet radiation and oxidative stress, leading to excessive reactive oxygen species production, activation of inflammatory mediators such as cyclooxygenase-2 and tumor necrosis factor α, disruption of skin barrier integrity, altered melanogenesis, and impaired hydration mechanisms.	Quercetin 3-O-*β*-D- glucuronide exerts anti-inflammatory, antioxidant, moisturizing, and anti-melanogenesis effects by reducing reactive oxygen species production, suppressing cyclooxygenase-2 and tumor necrosis factor alpha expression, activating nuclear factor erythroid 2-related factor 2 signaling, and modulating mitogen-activated protein kinase, activator protein-1, and nuclear factor kappa B pathways, leading to improved skin barrier and hydration-related gene expression.	In vitro experimental study using human keratinocytes (HaCaT), murine melanoma cells (B16F10), and human embryonic kidney cells exposed to ultraviolet radiation, hydrogen peroxide, and melanogenic stimuli.	Quercetin 3-O-*β*-D-glucuronide provides significant protection against inflammation, oxidative stress, pigmentation, and barrier dysfunction in skin cells, supporting its potential role as a multifunctional bioactive compound for skin protection and dermatological applications.	[73].
J.				
Oxidative stress-induced skin damage is characterized by excessive reactive oxygen species accumulation, mitochondrial dysfunction, apoptosis, dysregulation of antioxidant defenses, and altered gene expression in keratinocytes, contributing to inflammatory and pigmentary skin disorders.	Isorhamnetin protects keratinocytes from oxidative damage by reducing reactive oxygen species accumulation, enhancing antioxidant enzyme activity, preserving mitochondrial membrane potential, inhibiting apoptosis, and modulating oxidative stress-related gene expression through pathways including Wnt signaling.	In vitro experimental study using hydrogen peroxide-induced oxidative stress in human HaCaT keratinocytes combined with transcriptomic and bioinformatic analyses.	Isorhamnetin significantly attenuates oxidative stress-induced keratinocyte injury by restoring redox balance, mitochondrial function, and gene expression profiles, supporting its potential as a protective bioactive compound for oxidative stress-related skin disorders.	[74].
Atopic dermatitis is characterized by chronic inflammation, excessive oxidative stress, impaired keratinocyte migration, delayed wound healing, and dysregulated production of pro-inflammatory cytokines, contributing to persistent skin barrier dysfunction.	Quercetin attenuates atopic dermatitis-related pathology by reducing oxidative stress, suppressing pro-inflammatory cytokine expression, enhancing keratinocyte migration, and improving wound healing capacity through modulation of inflammatory and redox-sensitive signaling pathways.	In vitro experimental study using human keratinocyte atopic dermatitis models to assess inflammation, oxidative stress, and wound healing responses.	Quercetin significantly improves inflammatory status, oxidative balance, and wound healing in atopic dermatitis-like keratinocyte models, supporting its relevance as a bioactive flavonoid with therapeutic potential in inflammatory skin disorders.	[75].

## Data Availability

No new data were created or analyzed in this study.

## Supplementary Materials

The following supporting information can be downloaded at: https://www.mdpi.com/article/10.3390/medicina62040676/s1, PRISMA 2020 Checklist.

## Abbreviations

The following abbreviations are used in this manuscript:

SB	Sea buckthorn
